# Sexuality after Male-to-Female Gender Affirmation Surgery

**DOI:** 10.1155/2018/9037979

**Published:** 2018-05-27

**Authors:** J. Hess, A. Henkel, J. Bohr, C. Rehme, A. Panic, L. Panic, R. Rossi Neto, B. Hadaschik, Y. Hess

**Affiliations:** ^1^Department of Urology, University Hospital Essen, University Duisburg-Essen, Germany; ^2^Department of Urology, Kliniken Essen-Mitte, Essen, Germany; ^3^General Practice van Hal, Essen, Germany; ^4^Clinica Urologia, General Hospital Ernesto Simoes Filho, Salvador, Brazil

## Abstract

Male-to-Female (MtF) gender affirmation surgery (GAS) comprises the creation of a functional and aesthetic perineogenital complex. This study aimed to evaluate the effect of GAS on sexuality. We retrospectively surveyed all 254 MtF transsexual patients who had undergone GAS with penile inversion vaginoplasty at the Department of Urology, University Hospital Essen, Germany, between 2004 and 2010. In total, we received 119 completed questionnaires after a median of 5.05 years since surgery. Of the study participants, 33.7% reported a heterosexual, 37.6% a lesbian, and 22.8% a bisexual orientation related to the self-perceived gender. Of those who had sexual intercourse, 55.8% rated their orgasms to be more intensive than before, with 20.8% who felt no difference. Most patients were satisfied with the sensitivity of the neoclitoris (73.9%) and with the depth of the neovaginal canal (67.1%). The self-estimated pleasure of sexual activity correlated significantly with neoclitoral sensitivity but not with neovaginal depth. There was a significant correlation between the ease with which patients were able to become sexually aroused and their ability to achieve orgasms. In conclusion, orgasms after surgery were experienced more intensely than before in the majority of women in our cohort and neoclitoral sensitivity seems to contribute to enjoyment of sexual activity to a greater extent than neovaginal depth.

## 1. Introduction

Male-to-female (MtF) gender affirmation surgery (GAS) comprises the resection of all clearly defining features of male genitalia. The aim is the formation of a perineogenital complex in appearance and function as feminine as possible [1] with a sensitive clitoris to enable orgasms. GAS should be performed by a surgeon with specialized competence in genital reconstructive techniques [2]. The aim is to “create a perineogenital complex as feminine in appearance and function as possible” [1]. There is a broad agreement that GAS has a positive impact on gender dysphoria [3–13]. The inversion of penile skin is used by most gender surgeons. While some trans*∗* and gender nonconforming people do not require surgical therapy to express their preferred gender role and identity, others see GAS as a pivotal step to relieve their gender dysphoria [14]. GAS might reduce risk of stigmatization and discrimination in venues like swimming pools and health clubs or when dealing with authorities [2, 15]. Without doubt surgery has a positive effect on subjective wellbeing and sexual function [16–18].

Sexual orientation can change after GAS [19] but little is known about changes of orgasmic experience after GAS. Bartolucci et al. found a positive impact of cross gender hormone replacement therapy on sexual quality of life in transgender who had not undergone GAS yet [20]. However effects of GAS in this field remain unclear so far. This study aimed to evaluate the effect of GAS on sexuality and satisfaction with sexual life of MtF-transgender patients.

## 2. Material and Methods

### 2.1. Participants

Our study cohort comprised all 254 MtF patients who had undergone GAS with penile inversion vaginoplasty at the Department of Urology, University Hospital Essen, Germany, between 2004 and 2010, as has been previously reported [6]. Transsexualism was diagnosed by two independent mental healthcare professionals competent to work with gender dysphoric adults in accordance with 10th version of the International Classification of Diseases (ICD-10). All patients were contacted by mail using their last known address and asked if they would be willing to answer the questionnaire. In cases of invalid addresses the local residents' registration offices were contacted in order to reconsign a new questionnaire. Patients who had not sent back the questionnaire could not be followed up due to previous anonymization.

### 2.2. Statistics

Statistical calculation was performed using Statistical Package for the Social Sciences (SPSS 21.0). Fisher's exact test and Chi Square were used to compare categorical and ordinal variables in independent samples. The Mann–Whitney U test was used to compare satisfaction scale distribution of two independent samples. This nonparametric test was used in preference to the t-test because the Shapiro–Wilk test indicated that distribution was not normal. Spearman's correlation analysis was performed.

## 3. Results

In total, 119 completed questionnaires were received, all of which were included in the evaluation (response rate 46.9%). Due to anonymization of the questionnaires, it was not possible to obtain information on patients' ages. However, the average age of a comparable cohort of patients at our department between 1995 and 2008 [21] was 36.7 years (16 to 68 years). Not all patients completed the questionnaire, so for some questions the total number of responses was not 119. The results are given in absolute numbers and percentage in relation to total participants or number of answers. After a median of 5.05 years (standard deviation: 1.6 years; range: 1 to 7 years) since surgery, 67 participants (56.3% of the total cohort) did not encounter sexual intercourse on a regular basis at the time of questioning (which depicts 67.7% of those who answered that question). Twenty of the 119 patients (16.8%) did not answer this question. Of those who answered the question nearly a quarter (n = 24; 24.2%) reported a mean frequency of one to three times per month, seven (7.1%) stated a frequency of one to three times per week, and one woman (1.0%) stated a frequency of over three times per week. Time since GAS did not correlate with the frequency of intercourse and the self-rated intensity of orgasms. There was neither an association of the extent to which women felt female themselves nor with the degree to which they felt considered as women with time since surgery.

**Figure 1:**
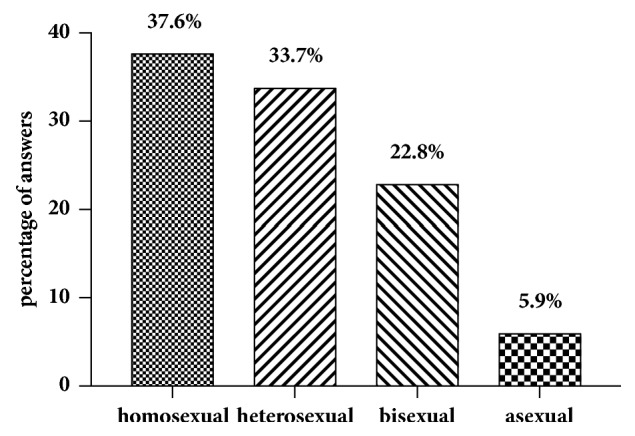
Sexual orientation related to the self-perceived gender.

**Figure 2:**
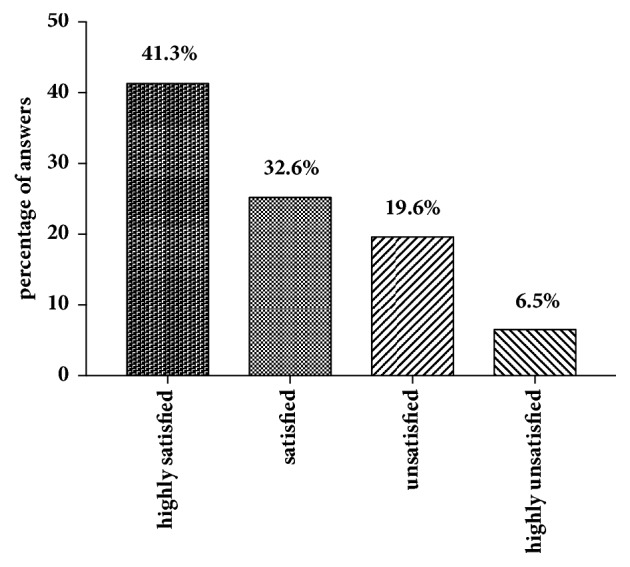
Satisfaction with neoclitoral sensitivity.

**Figure 3:**
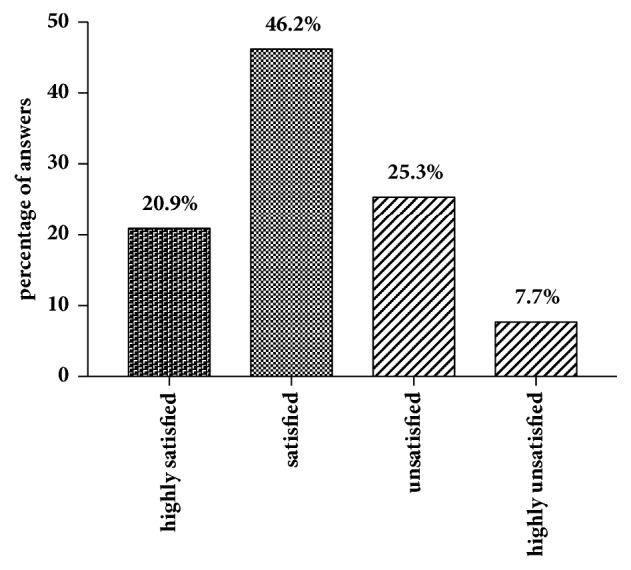
Satisfaction with neovaginal depth.

**Figure 4:**
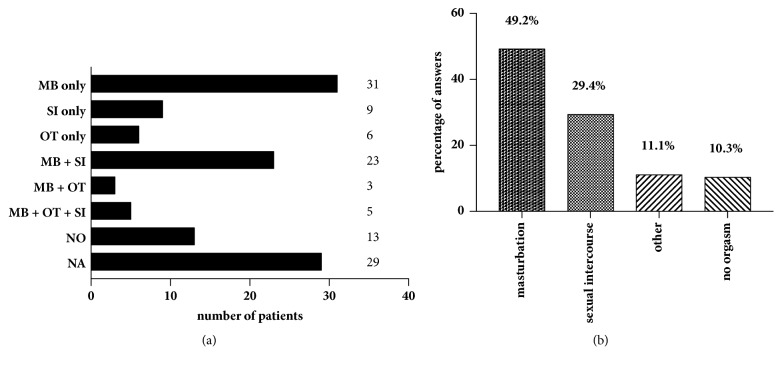
Modality as to how orgasms were achieved (multiple answers possible). (a) Absolute number of patients. MB = masturbation; SI = sexual intercourse; OT = other (not further specified); NO = no orgasm; NA = no answer. (b) Modality as percentage of answers.

**Figure 5:**
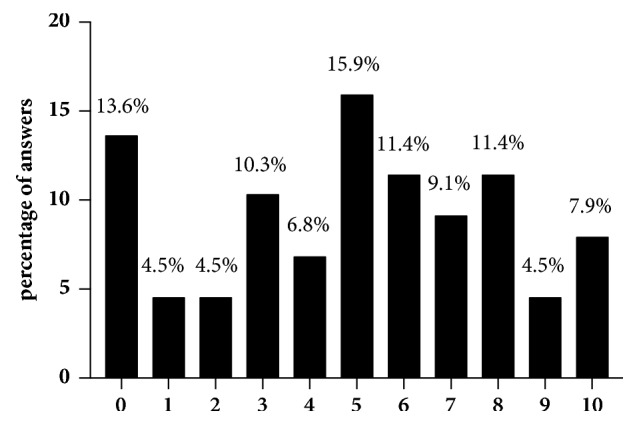
Patients' general satisfaction with their sex lives. Likert scale ranging from 0 (“very dissatisfied”) to 10 (“very satisfied”).

In our cohort, 18 (15.1% of all participants) patients refused to answer regarding sexual attraction related to the self-perceived gender. Of those who answered (n = 101), slightly more of the patients (n = 38; 37.6%) indicated a sexual attraction towards women than towards men (n = 34; 33.7%). 23 women (22.8%) were attracted by both men and women and six (5.9%) neither by men nor by women (Figure 1). In total, 38 subjects (41.3%) were highly satisfied, 30 (32.6%) were satisfied, 18 (19.6%) were not satisfied, and six (6.5%) were highly unsatisfied with the sensitivity of the neoclitoris (Figure 2). This question was not answered by 27 individuals (22.7% of all participants). When asked how satisfied the women were with the depth of the neovaginal canal, 19 were very satisfied (20.9%), 42 (46.2%) were satisfied, 23 (25.3%) were unsatisfied, and seven (7.7%) were very unsatisfied, with 28 (23.5% of all participants) not answering the question (Figure 3). We asked our patients whether it was easy to get sexually aroused. In total 91 women responded to this question, and about a quarter (n = 28; 23.5% of all participants) declined to answer. Of these 91 women 22 (24.2%) stated that this was always easy; for 43 (47.3%) it was mostly easy; for 15 (16.5%) it was seldom easy; and for eleven women (12.1%) it was never easy to get sexually aroused. The modality as to how orgasms were achieved is shown in Figure 4(a) (absolute numbers of patients; n = 119) and Figure 4(b) (percentages expressed in relation to total answers; n = 126). The majority of participants achieved an orgasm with masturbation, followed by sexual intercourse and “other” not further specified sexual practices. 29 women (24.4% of all participants) did not answer that question. Of those who answered that question (n = 77), 43 women (55.8%) quoted that orgasms were more intense after GAS compared with those experienced before surgery, 18 (23.4%) women stated that it was less intense than before, and 16 (20.8%) felt no difference. Frequency of achieved orgasms changed in our cohort after GAS. Of all 119 patients 41 (34.5%) refused to answer that question. Of the residual 78 women 41 (52.6%) indicated that orgasms were achieved less frequently, 21 women (26.9%) reported more frequent orgasms, and for 16 women (20.5%), frequency did not change. In order to gather information on patients' general satisfaction with their sex lives, they were asked to place themselves on a Likert scale ranging from 0 (“very dissatisfied”) to 10 (“very satisfied”). Nearly a quarter of participants either selected scores from 0 to 3 (n = 29; 24.4%), from 4 to 6 (n = 30; 25.2%), or from 7 to 10 (n = 29; 24.4%) or refused to answer (n = 31; 26.1%).  Figure 5 shows a detailed illustration. We received feedback regarding pleasure of sexual activity from 88 women (73.9%). Of these respondents 31 (35.2%) stated that sexual activity was always pleasurable; 44 (50.0%) said it was sometimes pleasurable and 13 (14.8%) never felt pleasure with sexual activity. In our cohort, there was a significant correlation between the ease of getting sexually aroused and the ability to achieve an orgasm (r_s_ = 0.616, p = 0.01). The better the sexual arousal, the easier it was to achieve an orgasm. The correlation between arousal and sensitivity of the neoclitoris was less distinctive but still significant (r_s_ = 0.506, p = 0.01). The self-estimated pleasure of sexual activity was significantly correlated with the sensitivity of the neoclitoris (r_s_ = 0.508, p = 0.01) but not with the depth of the neovaginal canal (r_s_ = 0.198, p = 0.079); i.e., neoclitoral sensitivity seems to contribute to the enjoyment of sexual activity to a greater extent than the depth of the neovagina.

## 4. Discussion

Overall, subjective satisfaction rates can be expected to be 80% and higher after GAS [22]. Löwenberg reported a general satisfaction with the outcome of GAS to be even over 90% [10]. Studies often stress the emphasis on functional or aesthetic aspects after GAS [5–7, 23–25] or, at best, on sexual quality of life before GAS [20, 26]. To our best knowledge, this is the first study placing a particular focus on sexual life after MtF GAS.

In our study, sexual attraction was referred to the self-perceived sexual identity on the basis of self-identification. Accordingly, we used the term “heterosexual” or “homosexual” when participants reported on sexual attraction towards men (natal men as well as transmen) and women, respectively. Due to the existing stigmatization of homosexual and lesbian individuals in a heteronormative community or to patients' wish for social desirability, it is possible that reports on the prevalence of homosexuality (gay and lesbian) are underestimations. A representative study with over 14.000 men and women in Germany reported on a prevalence of 4% of men and 3% of women who self-identified as “gays”. Another 9% of male and 20% of female heterosexual participants felt sexually attracted by the same sex without identifying themselves as gay [27]. International surveys found a prevalence of homosexuality in up to 3% with regional and age-dependent variations [28–32]. In our study, the percentage of homosexuality (gay and lesbian) related to self-perceived gender was much higher. This could be because the interviewees knew the interrogators well, had generally revealed their sexual orientation beforehand, and had no fear of societal stigmatization. There is also the possibility that the rate of homo- and bisexuality is, in fact, higher in transsexuals compared with nontranssexuals. [33] Lawrence found a change in predominant sexual attraction in 232 MtF transsexuals before and after genital reassignment [19]. In her study, 54% and 25% of participants reported a gynephile orientation before and after surgery, respectively. Androphilic orientation changed from 9% preoperatively to 34% postoperatively. Regarding asexuality, we followed the definition of Prause and Graham who found that asexuality is defined to be a lack of sexual interest or desire, rather than a lack of sexual experience [34]. In our cohort, in total 6% of the women self-identified as asexual. Bogaert reported on approximately 1% asexual individuals of a total sample size of over 18.000 (nontranssexual) British residents, with more women being asexual than men [35]. He found both biological and psychosocial factors contributing to the development of asexuality. Prause and Graham found significantly lower sexual arousability and lower sexual excitation in asexual individuals with a prevalence of 4% [34]. A reduced sensitivity of the neoclitoris could therefore be a prognostic factor for asexuality. Our results support this assumption. The sensitivity of the neoclitoris correlated with the ability of sexual arousal and achieving an orgasm, as well as with the self-estimated pleasure of sexual activity. In our cohort, satisfaction with the sensitivity of the neoclitoris was higher than with the depth of the neovaginal canal. This could be due to the time of questioning, which was a median of 5.05 years after GAS. While neoclitoral sensitivity is unlikely to diminish, it is more likely that the neovaginal canal shrinks over time. Of the subjects 6% reported a stenosis of the neovagina and 45% a loss of initial neovaginal depth [25]. The longer the period after GAS is, the more prevalent the stenosis of the neovaginal canal seems to be [36]. Ineffective dilatation of the neovaginal canal is obviously a key factor contributing to neovaginal stenosis. Over half of all patients (58%) do not use vaginal dilators appropriately, which is a major reason for this kind of long-term complication [36].

Postsurgical sexuality plays an important role in overall satisfaction and depends substantially on the functionality of the neovagina [5, 6]. Satisfaction with functionality ranges between 56% and 84% [7, 9, 10, 37, 38]. Previously, we reported a satisfaction rate with functionality, including satisfaction with depth and breadth of the neovagina and the satisfaction with penetration or intercourse, to be 72% (“very satisfied” and “satisfied”) or 91% (including also “mostly satisfied”) [6]. The self-reported enjoyment of sexual activity correlated significantly and to a greater extent with neoclitoral sensitivity than with neovaginal dimensions, which was not significant. Though genital dimensions were not surveyed in our study, penile size often exceeds the depth of the vaginal canal in natal women without causing problems with, or pain during, sexual intercourse. However in contrast to a skin derived vaginal canal of transgender women the vagina of natal women is able to expand 2.5 to 3.5 cm in length when sexually stimulated [39]. Neoclitoral sensitivity is usually assessed by means simply of asking the women and can be biased by the patients' wish for social desirability. In this retrospective study we could not rule this out. However, we previously introduced a measurement tool to assess semiquantitatively the sensitivity with a customary brush and a tuning fork [40] which could be used for future studies on this topic. Though the rate of women, who were able to achieve an orgasm, was lower in the present study than in an earlier cohort from our department [9], our data aligns well with comparable studies of a similar size [11, 19, 41–43]. Interestingly, Dunn et al. found a similar rate of natal women who were unsure or not able to achieve an orgasm during intercourse (16%) or masturbation (14%) [44]. In total 55.8% of the women in our study rated their orgasms postoperatively as more intense than before surgery, one in five women (20.8%) felt no difference, and 23.4% reported less intense orgasms after surgery. These results are roughly in line with a study by Buncamper et al. [45]. Since it is very unlikely that handling of the neurovascular bundle during surgery will make the neoclitoris more sensitive than the glans penis was before, a possible explanation could be that postoperative patients were able to experience orgasm for the first time in a body that matched their perception. Furthermore, a decline in sexual desire after sex reassignment therapy (hormonal and surgical) could contribute to an altered orgasmic experience [46]. Interestingly, in their systematic review, Guillamon et al. reported on results of three longitudinal studies showing a transformation in the brain morphology of MtF after initiation of cross sex hormonal therapy towards a more female morphology [47]. Moreover, receiving hormonal treatment was one of the factors related to a better subjective perception of sexual quality of life [20]. Rolle et al. registered a cerebral modification after sex reassignment in fifteen MtF transsexual individuals towards a more female cognitive response [48]. It is unclear whether this could explain differences in subjective orgasm experience before and after GAS. Further prospective studies with a larger sample size are needed to validate this preliminary aspect.

## 5. Limitations

The study was limited by its retrospective character with a response rate below 50%. Suicide is a very unlikely reason for nonparticipation since the suicide rate after successful GAS is not higher than in the general population [49]. However, contacting trans-female patients for long-term follow-up is generally difficult [3, 37, 50–54] particularly in countries like Germany where there is no central registration. Another reason is that patients often move following successful surgery [5]. Response rates to surveys in retrospective research in this field are between 19% [54] and 79% [55]. With 49%, Löwenberg et al. achieved a similar response rate in a follow-up inquiry of a comparable cohort [10]. Another bias could be that the answers represent patients' wishes for social desirability, rather than the reality of their situation. However, this cannot be verified retrospectively.

## 6. Conclusion

To our best knowledge, this was the first study to survey sexuality after MtF GAS in a very detailed way. In the majority of women, orgasms after surgery were experienced more intense than before. In our cohort, neoclitoral sensitivity seems to contribute to enjoyment of sexual activity to a greater extent than the depth of the neovaginal canal.
